# CONTENTS OF VOLUME 15, 2022

**DOI:** 10.3400/avd.cv.22-01000

**Published:** 2022-12-25

## No. 1

### REVIEW ARTICLE

**1 Isolated Superior Mesenteric Artery Dissection: A Novel Etiology and a Review**

Rakan Nasser Eldine, MD, Hassan Dehaini, MD, Jamal Hoballah, MD, and Fady Haddad, MD, FACS

### ORIGINAL ARTICLES

**8 Treatment Strategies for Improving the Surgical Outcomes of Ruptured Abdominal Aortic Aneurysm: Single-Center Experience in Japan**

Yasumi Maze, MD, PhD, Toshiya Tokui, MD, PhD, Masahiko Murakami, MD, Teruhisa Kawaguchi, MD, Ryosai Inoue, MD, Bun Nakamura, MD, Koji Hirano, MD, PhD, Shuji Chino, MD, Ken Nakajima, MD, and Noriyuki Kato, MD, PhD

**14 The Patency of Tibial/Peroneal Arteries Affects the Increment of Regional Tissue Saturation of Oxygen in Each Angiosome after Superficial Femoral Artery Revascularization**

Naoki Unno, MD, PhD, Kazunori Inuzuka, MD, PhD, Naoto Yamamoto, MD, PhD, Masaki Sano, MD, PhD, Kazuto Katahashi, MD, PhD, Takafumi Kayama, MD, Tatsuro Yata, MD, Yuta Yamanaka, MD, Hajime Tsuyuki, MD, Yusuke Endo, MD, Nozomu Ishikawa, MD, Ena Naruse, CVT, Masatsugu Niwayama, PhD, and Hiroya Takeuchi, MD, PhD

**22 Ultrasound-Guided Thrombin Injection for Postcatheterization Pseudoaneurysms and Its Extended Indications**

Hirotsugu Ozawa, MD, Takao Ohki, MD, PhD, Kenjiro Kaneko, MD, Masamichi Momose, MD, and Shigeki Hirayama, MD

**29 Buerger Disease: Pathological Changes in Elderly Patients**

Takehisa Iwai, MD, PhD, Hiroko Kume, MD, PhD, Shinya Koizumi, MD, Kenichi Sakurazawa, MD, PhD, Kaori Honma, MHS, Hiroko Ogasawara, BS, Tamiko Takemura, MD, PhD, Mitsuhiro Kishino, MD, PhD, and Tomoko Kagayama, MHS

**37 Bicuspidalization of the Native Tricuspid Aortic Valve: A Porcine in Vivo Model of Bicuspid Aortopathy**

Naoyuki Kimura, MD, PhD, Ryo Itagaki, MD, PhD, Masanori Nakamura, PhD, Alimuddin Tofrizal, MD, PhD, Megumi Yatabe, MS, Takamichi Yoshizaki, MD, PhD, Ryo Kokubo, CCP, Shuji Hishikawa, MD, PhD, Satoshi Kunita, MD, PhD, Hideo Adachi, MD, PhD, Yoshio Misawa, MD, PhD, Takashi Yashiro, MD, PhD, and Koji Kawahito, MD, PhD

### CASE REPORTS

**45 Internal Iliac Artery Aneurysm Ruptures with No Visualized Endoleak 2 Years after Endovascular Repair**

Ayumi Harada, MD, Koichi Morisaki, MD, PhD, Shun Kurose, MD, Shinichiro Yoshino, MD, Sho Yamashita, MD, Tadashi Furuyama, MD, PhD, and Masaki Mori, MD, PhD

**49 A Case of Common Carotid Artery Occlusion: A Unique Pattern of Occlusion and Lessons Learned**

Thilina Gunawardena, MBBS, MD, MRCS, Manujaya Godakandage, MBBS, MD, MRCS, Sachith Abeywickrama, MBBS, MD, MRCS, Balasubramaniyam Saseekaran, MBBS, MD, MRCS, Rezni Cassim, MBBS, MS, MRCS, and Mandika Wijeyaratne, MBBS, MD, FRCS

**53 Prevention of Buttock Claudication by Preserving Antegrade Bilateral Superior Gluteal Arterial Blood Flow in EVAR for Aorto-Iliac Aneurysm Accompanied by Bilateral Internal Iliac Artery Aneurysms**

Yuta Tajima, MD, PhD, Hitoshi Goto, MD, PhD, Daijiro Akamatsu, MD, PhD, Fukashi Serizawa, MD, PhD, Shunya Suzuki, MD, PhD, Shinichiro Horii, MD, PhD, Norinobu Ogasawara, MD, Hirokazu Takahashi, MD, Yohei Nagaoka, MD, and Takashi Kamei, MD, PhD

**58 Successful Surgical Repair of Type A Acute Aortic Dissection in a Patient with Vascular Ehlers–Danlos Syndrome**

Kousuke Mori, MD, Hirohito Ishii, MD, Shuhei Sakaguchi, MD, Daichi Sakurahara, MD, Ayaka Iwasaki, MD, and Koji Furukawa, MD, PhD

**62 Impact of High-Resolution Epiaortic Ultrasonographic Imaging on Evaluating Aortic Wall Pathology**

Soichiro Henmi, MD, So Izumi, MD, Ryoichi Mizoue, MD, Yutaka Okita, MD, Kenji Okada, MD, and Takuro Tsukube, MD

**64 Acute Limb Ischemia Caused by Embolus of Primary Lung Cancer Complicating Trousseau’s Syndrome**

Chiharu Tanaka, MD, PhD, Fumio Sakamaki, MD, PhD, Hidekazu Furuya, MD, Masaomi Yamaguchi, MD, Kazuo Kanabuchi, MD, PhD, and Kenji Kuwaki, MD, PhD

**68 Takayasu Arteritis Complicated by Ischemic Colitis: A Case Report**

Shigeyuki Yamashita, MD, Kanetsugu Nagao, MD, Toshio Doi, MD, Shigeki Yokoyama, MD, Akio Yamashita, MD, Kazuaki Fukahara, MD, and Naoki Yoshimura, MD

**72 Stent-Graft Removal and Extra-Anatomical Bypass for the Treatment of Stent-Graft Infection after Endovascular Aneurysm Repair**

Naoya Kuriyama, MD, Atsuhiro Koya, MD, Shinsuke Kikuchi, MD, PhD, Daiki Uchida, MD, PhD, and Nobuyoshi Azuma, MD, PhD

**77 Recurrent Mural Thrombosis of the Ascending Aorta in a Patient with Antiphospholipid Syndrome**

Ryohei Otsuka, MD, Shunei Saito, MD, PhD, Toshikuni Yamamoto, MD, Tsukasa Ohno, MD, Akio Koyama, MD, Hirofumi Morimae, MD, Masahiro Matsushita, MD, PhD, Kaori Yokota, MD, Ken Miyahara, MD, PhD, and Akio Matsuura, MD, PhD

### HOW TO DO IT

**81 Tailor-Made Tapering Grafts for Large-Neck Aorta**

Takuro Shirasu, MD, PhD, Masaru Kimura, MD, PhD, Takanori Kaneko, MD, Takatoshi Furuya, MD, PhD, Kaito Fukuda, MD, Motoki Nagai, MD, and Yukihiro Nomura, MD

**85 Erratum**

## No. 2

### REVIEW ARTICLE

**87 Superior Vena Cava Syndrome and Wallstent: A Systematic Review**

Ali Kordzadeh, MBBS, MSc, MD, VA-BC, FEBS, FEVBS, Alan Askari, MBChB, MSc, DIC, PhD, MRCS FRCS, Muhammad A. Hanif, MRCS, FRCR, and Vijay Gadhvi, MBBS, MRCS, MSc, FRCS

### ORIGINAL ARTICLES

Selection from the Japanese Journal of Phlebology 2020

**94 Water Distribution Changes in Complex Decongestive Treatment for Leg Lymphedema: Quantitative Evaluation by Direct Segmental Multi-Frequency Bioimpedance Analysis**

Masahiro Toshima, MD, PhD and Yoshihisa Morino, RPT

**101 Clinicopathological Characteristics of Cancer-Associated Venous Thromboembolism (CAT-VTE) from a Medicolegal Autopsy**

Ayako Ro, MD, PhD, Norimasa Kageyama, BBA, and Toshiji Mukai, MD, PhD

**107 Characteristics and Prognostic Factors of Venous Thromboembolism in Cancer Patients**

Hajime Tsuyuki, MD, Naoto Yamamoto, MD, Naoki Unno, MD, PhD, Kazunori Inuzuka, MD, PhD, Masaki Sano, MD, PhD, Kazuto Katahashi, MD, PhD, Tatsuro Yata, MD, PhD, Takafumi Kayama, MD, PhD, Yuta Yamanaka, MD, Yusuke Endo, MD, and Hiroya Takeuchi, MD, PhD

### ORIGINAL ARTICLES

**113 Management of Acute Limb Ischaemia Due to COVID-19 Induced Arterial Thrombosis: A Multi-Centre Indian Experience**

Natarajan Sekar, MS, MCh, DSc, FRCS, Jithin Jagan, MS, MCh, Arunagiri Viruthagiri, MS, MCh, Nedounsejiane Mandjiny, MS, MCh, and Karthikeyan Sivagnanam, MS, DNB (vascular)

**121 Relevance of Non-Bridging Therapy with Heparin during Temporary Interruption of Direct Oral Anticoagulants in Patients with Cancer-Associated Venous Thromboembolism**

Takuya Oyakawa, MD, Masafumi Fukumitsu, MD, PhD, Aya Ebihara, MD, PhD, and Taro Shiga, MD, PhD

**126 Efficacy of Early Closed Toe Amputation for Toe Ulcers with Suspected Osteomyelitis after Revascularization for Chronic Limb-Threatening Ischemia**

Tsunehiro Shintani, MD, PhD, Sachi Suzuki, MD, Naoya Kikuchi, MD, Takumi Ariya, MD, Kayoko Natsume, MD, PhD, Kazuhiro Ookura, MD, PhD, Jun Okui, MD, Yasunori Sato, PhD, and Hideaki Obara, MD, PhD

### CASE REPORTS

**134 Successful Open Thoracoabdominal Aortic Repair in a Patient with Severe Aorto-Iliac Occlusive Disease: A Rare Case Report**

Takanori Tsujimoto, MD, Tatsuro Asada, MD, PhD, Akitoshi Yamada, MD, PhD, and Kunio Gan, MD, PhD

**138 Femoropopliteal Bypasses with Varicose Great Saphenous Vein**

Sosei Kuma, MD, PhD, Kazuomi Iwasa, MD, PhD, Takuya Matsumoto, MD, PhD, and Hideaki Uchiyama, MD, PhD

**142 A Case of Alport Syndrome Associated with Recurrent Stanford Type B Aortic Dissections**

Miki Takeda, MD, Tadanori Minagawa, MD, PhD, Wakiko Hiranuma, MD, Takayuki Matsuoka, MD, Takuya Shimizu, MD, PhD, and Shunsuke Kawamoto, MD, PhD

**146 Transfusion-Related Acute Lung Injury Type I Immediately after Open Surgical Repair for Abdominal Aortic Aneurysm**

Kota Shimizu, MD, Michihisa Umetsu, MD, PhD, Hitoshi Goto, MD, PhD, Takuya Fujimine, MD, PhD, Daijirou Akamatsu, MD, PhD, and Takashi Kamei, MD, PhD

**150 Traumatic Superficial Femoral Arteriovenous Fistula with Pulsatile Mass and Leg Pain 60 Years after Stabbing Injury**

Kazuki Takahashi, MD, Shinsuke Kikuchi, MD, PhD, Ai Tochikubo-Suzuki, MD, Yuri Yoshida, MD, PhD, Daiki Uchida, MD, PhD, Atsuhiro Koya, MD, Kazuya Kato, MD, PhD, and Nobuyoshi Azuma, MD, PhD

**154 Endovascular and Endoscopic Treatment for Primary Aortoduodenal Fistula: A Case Report**

Kazuki Noda, MD, Koki Yokawa, MD, Naoki Dan, MD, and Hitoshi Matsuda, MD

**157 Extended Aortic Repair for Acute Type A Aortic Dissection with Rupture and Malperfusion Complicated with Ehlers-Danlos Syndrome**

Koki Maekawa, MD, Toshiki Fujiyoshi, MD, PhD, Masaki Kano, MD, Yu Nakano, MD, Ryumon Matsumoto, MD, and Hitoshi Ogino, MD, PhD

**161 Endovascular Therapy for Isolated Brachiocephalic Pseudoaneurysm**

Daisuke Arima, MD, Kazuchika Suzuki, MD, PhD, Yumi Kando, MD, Satoshi Akuzawa, MD, PhD, and Naoyuki Ishigami, MD

## No. 3

### REVIEW ARTICLE

**165 Current Clinical Implications of Frailty and Sarcopenia in Vascular Surgery: A Comprehensive Review of the Literature and Consideration of Perioperative Management**

Hiroshi Furukawa, MD, PhD

### ORIGINAL ARTICLE

Selection from Japanese Journal of Vascular Surgery 2021

**175 Procedure and Aortic Remodeling Effects of Entry Closure with Stentgraft for Type B Aortic Dissection: Comparison between the Patients with Narrow True Lumen and Those with Aneurysmal Dilated False Lumen**

Atsushi Aoki, MD, PhD, Kazuto Maruta, MD, PhD, Tomoaki Masuda, MD, PhD, and Tadashi Omoto, MD, PhD

### ORIGINAL ARTICLE

**186 Lipoprotein(a) is a Promising Residual Risk Factor for Long-Term Clinical Prognosis in Peripheral Arterial Disease**

Kimimasa Sakata, MD, PhD, Hisao Kumakura, MD, PhD, Ryuichi Funada, MD, PhD, Yae Matsuo, MD, PhD, Kuniki Nakashima, MD, PhD, Toshiya Iwasaki, MD, and Shuichi Ichikawa, MD, PhD

### CASE REPORTS

**193 Iatrogenic Arteriovenous Fistula of Subclavian Artery to Vertebral Vein with Perimedullary Vein Reflux**

Koji Sato, MD, PhD, Yasushige Shingu, MD, PhD, Masato Fusegawa, MD, Takahiro Ishigaki, MD, and Satoru Wakasa, MD, PhD

**197 Surgical Treatment of a Giant Popliteal Venous Aneurysm and Arteriovenous Fistula on the Adjacent Femoral Vein and Its Postoperative Findings**

Atsushi Hiromoto, MD, Shun-ichiro Sakamoto, MD, PhD, Yusuke Motoji, MD, Ryosuke Amitani, MD, Takako Yamaguchi, RN, Kenji Suzuki, MD, PhD, Hiromasa Yamashita, MD, Makoto Watanabe, MD, Eitaro Kodani, MD, PhD, and Yosuke Ishii, MD, PhD

**201 Wound Healing on the Cutting Plane of Ankle Bones after Incomplete Revascularization for Chronic Limb-Threatening Ischemia in an Elderly Patient: A Case Report**

Shinsuke Kikuchi, MD, PhD, Daiki Uchida, MD, PhD, Kazuki Takahashi, MD, Yuri Yoshida, MD, PhD, Ai Tochikubo-Suzuki, MD, Tomoki Nakatsu, MD, Mineko Higuchi, RN, Nobuyoshi Azuma, MD, PhD, and Kazuya Kato, MD, PhD

**206 Surgical Management of Perigraft Seroma by Graft Wrapping with Local Hemostatic Agents: A Case Report**

Hiroshi Furukawa, MD, PhD, Noriyasu Masuda, MD, PhD, and Kazuhiko Uwabe, MD, PhD

### ANNUAL REPORT

**210 2019 JAPAN Critical Limb Ischemia Database (JCLIMB) Annual Report**

The Japanese Society for Vascular Surgery JCLIMB Committee, NCD JCLIMB Analytical Team

**239 Erratum**

## No. 4

### REVIEW ARTICLE

Therapeutic Angiogenes Update

**241 Therapeutic Angiogenesis Using Autologous CD34-Positive Cells for Vascular Diseases**

Yasuyuki Fujita, MD, PhD and Atsuhiko Kawamoto, MD, PhD

### REVIEW ARTICLE

**253 Role of Simulated Training for Carotid Endarterectomy: A Systematic Review**

Nadeem A. Siddiqui, MBBS, FCPS, FACS, MSc, Ammar Pirzada, MBBS, FCPS, Shoaib Badini, MBBS, FCPS, and Fareed A. Shaikh, MBBS, MRCSEd, FCPS-GS, FCPS-Vascular Surgery

### ORIGINAL ARTICLES

Selection from the Journal of Japanese College of Angiology 2021

**260 Computational Fluid Dynamics Analysis of Blood Flow Changes during the Growth of Saccular Abdominal Aortic Aneurysm**

Masanori Murakami, MD, PhD, Fei Jiang, PhD, Nobuyasu Kageyama, MEng, and Xian Chen, PhD

**268 Useful Test for Classification of Cerebral Infarction at Hospital Specializing in Neurosurgery**

Hirotaka Yoshida, MD and Kazutoshi Nishitani, MD, PhD

**275 Venous Stenting for Postthrombotic Iliocaval Venous Obstructive Disease: Clinical Efficacy and Mid-term Outcomes**

Yuji Hoshino, MD, PhD and Hiroyoshi Yokoi, MD, PhD

### ORIGINAL ARTICLES

**282 Management of Arteriovenous Graft Infection**

Yoichi Hisata, MD, Taku Inoue, MD, Yuichi Tasaki, MD, Tomohiro Odate, MD, and Takafumi Yamada, MD

**289 Validation of Task-Specific Rating Scale for Open Balloon Catheter Arterial Embolectomy: An Assessor-Blinded Quasi-Experimental Pilot Study**

Nadeem Ahmed Siddiqui, FRCS, Shiraz Hashmi, Iram Naz, MD, and Ziad Sophie, MD

**295 Impact of the Isolated Cerebral Perfusion Technique for Aortic Arch Aneurysm Repair in Patients with a Shaggy Aorta**

Kayo Sugiyama, MD, PhD, Hirotaka Watanuki, MD, PhD, Masato Tochii, MD, PhD, Yasuhiro Futamura, MD, Koki Ishizuka, MD, and Katsuhiko Matsuyama, MD, PhD

**301 Pulse Amplitude Measured with a Portable Laser Doppler Flowmeter Is Useful for Screening of Dialysis Patients for Peripheral Arterial Disease: An Observational Study**

Makoto Saito, AS, Hiroomi Jingu, AS, Hidefumi Osawa, MD, Yusuke Oyama, MD, Toshiyuki Tanaka, MD, Akihiko Shiono, MD, and Masami Machida, MD

**308 Midterm Results of Thoracic Endovascular Aortic Repair with Reentry Closure for Chronic Type B Aortic Dissection with Aneurysmal Dilatation**

Kiyoshi Chiba, MD, PhD, Hiroshi Nishimaki, MD, PhD, Yukihisa Ogawa, MD, PhD, Masahiro Tomita, MD, Ryuji Nakamura, MD, Satoshi Kinebuchi, MD, PhD, Shota Kita, MD, PhD, Masahide Komagamine, MD, PhD, Kan Nawata, MD, PhD, Masahide Chikada, MD, PhD, and Takeshi Miyairi, MD, PhD

**317 Anticoagulation Therapy for Pregnancy-associated Thrombosis: A Retrospective Observational Study**

Michihisa Umetsu, MD, PhD, Daijirou Akamatsu, MD, PhD, Fukashi Serizawa, MD, PhD, Yuta Tajima, MD, PhD, Shunya Suzuki, MD, PhD, Shinichiro Horii, MD, PhD, Norinobu Ogasawara, MD, PhD, Hirokazu Takahashi, MD, Yohei Nagaoka, MD, Kota Shimizu, MD, Shunsaku Kimura, MD, Munetaka Hashimoto, MD, PhD, Hitoshi Goto, MD, PhD, Tetsuo Watanabe, MD, PhD, and Takashi Kamei, MD, PhD

### CASE REPORTS

**324 A Case Series of Secondary Aortoenteric Fistula after Open Aortic Aneurysm Repair: Timing and Technique of Surgery**

Kentaro Kasa, MD, Hiroshi Hirukawa, MD, Shintaro Fukuda, MD, Fuyuki Asami, MD, Masatake Katsu, MD, Kazuo Yamamoto, MD, and Shinpei Yoshi, MD

**329 Embolization of Deep Femoral Artery Aneurysm with a Ligated Proximal Artery Using the Direct Percutaneous Puncture Technique**

Kohei Hamamoto, MD, PhD, Takao Nonaka, MD, Koichi Tamai, MD, Emiko Chiba, MD, PhD, Noriko Oyama-Manabe, MD, PhD, Yohsuke Suyama, MD, PhD, Sadahiro Watanabe, MD, Eiko Hyoe, MD, and Hiroshi Shinmoto, MD, PhD

**333 Successful Crossover Bypass Using a Lateral Femoral Circumflex Artery as an Outflow Vessel for Indirect Revascularization in Critical Limb Ischemia: A Case Report**

Kei Akiyoshi, MD, Mamoru Arakawa, MD, PhD, Harunobu Matsumoto, MD, Koichi Adachi, MD, and Hiroko Nakata, MD

**337 Endovascular Repair for Abdominal Aneurysm with Concomitant Aortoiliac Vein Fistula Diagnosed by Four-Dimensional Computed Tomography**

Chiharu Tanaka, MD, PhD, Hidekazu Furuya, MD, Shunsuke Kamei, MD, Satoshi Suda, MD, and Masaomi Yamaguchi, MD

**341 Longterm Followup of a Pediatric Patient with Congenital Abdominal Aortic Aneurysm with Coarctation**

Masahide Chikada, MD, Kiyoshi Chiba, MD, Kan Nawata, MD, Masahiro Tomita, MD, Ryuji Nakamura, MD, Satoshi Kinebuchi, MD, Shota Kita, MD, Masahide Komagamine, MD, Misa Kogo, MD, Hiroshi Nishimaki, MD, and Takeshi Miyairi, MD

**344 Intimal Sarcoma after Endovascular Abdominal Aortic Aneurysm Repair**

Masaki Komatsu, MD, Kazuki Naito, MD, Shuji Chino, MD, Haruki Tanaka, MD, Hajime Ichimura, MD, PhD, Takateru Yamamoto, MD, Ko Nakahara, MD, Megumi Fuke, MD, PhD, Yuko Wada, MD, PhD, and Tatsuichiro Seto, MD, PhD

**348 Assessment of Pseudocoarctation of the Aorta with Saccular Aneurysms by Four-Dimensional Flow Magnetic Resonance Imaging and Histological Analysis**

Hiromasa Ito, MD, Yoshito Ogihara, MD, PhD, Masaki Ishida, MD, PhD, Hisato Ito, MD, PhD, Kyoko Imanaka-Yoshida, MD, PhD, and Kaoru Dohi, MD, PhD

**352 Cardiopulmonary Bypass Surgery in a Patient with Unexpected Heparin-induced Thrombocytopenia**

Atsushi Miyagawa, MD, Koichi Yuri, MD, PhD, Mitsunori Nakano, MD, Daigo Shinoda, MD, and Jun Makino, MD

**356 Reviewers Index**

**358 Authors Index**

**361 Contents of Volume 15, 2022**

**368 Best Cited Articles 2022**

